# Associations between interoceptive sensitivity, intuitive eating, and body mass index in patients with anorexia nervosa and normal‐weight controls

**DOI:** 10.1002/erv.2676

**Published:** 2019-04-09

**Authors:** Anna Richard, Adrian Meule, Claudio Georgii, Ulrich Voderholzer, Ulrich Cuntz, Frank H. Wilhelm, Jens Blechert

**Affiliations:** ^1^ Department of Psychology University of Salzburg Salzburg Austria; ^2^ Centre for Cognitive Neuroscience University of Salzburg Salzburg Austria; ^3^ Schoen Clinic Roseneck Prien am Chiemsee Germany; ^4^ Department of Psychiatry and Psychotherapy University Hospital Freiburg Freiburg Germany; ^5^ Department of Psychiatry and Psychotherapy University Hospital Munich Munich Germany; ^6^ Paracelsus Medical University Salzburg Austria; ^7^ Division of Clinical Psychology, Psychotherapy, and Health Psychology, Department of Psychology University of Salzburg Salzburg Austria

**Keywords:** anorexia nervosa, body mass index, heartbeat perception, interoceptive sensitivity, intuitive eating, weight gain

## Abstract

Reduced perception of bodily signals and low levels of intuitive eating have been reported in patients with anorexia nervosa (AN) compared with normal‐weight individuals. However, findings have been inconsistent and treatment progress might account for some of these inconsistencies. Thirty‐seven inpatients with AN and 39 normal‐weight controls completed a heartbeat perception task and the Intuitive Eating Scale–2. Patients with AN reported lower intuitive eating than controls, whereas interoceptive sensitivity did not differ between groups. Higher interoceptive sensitivity was related to higher intuitive eating across both groups. In patients with AN, both higher interoceptive sensitivity and intuitive eating correlated with the number of days in the hospital and with higher body mass index (BMI), when controlling for BMI at admission. These relationships suggest that interoceptive sensitivity and intuitive eating improve during treatment. Future research should determine whether these improvements promote weight gain or follow it.

## 1 INTRODUCTION

Interoception refers to a sense of the body in the brain based on afferent feedback of inner organs and is, therefore, fundamental for a range of adaptive behaviors (e.g., recognizing emotions or signs of hunger/fullness; Cameron, 2001; Craig, 2002). Sensitivity for interoceptive processes may be deficient in anorexia nervosa (AN) and an inability to perceive and process bodily signals adequately has been considered as a precursor and maintaining factor of AN (Bruch, 1962). Therefore, research in the past decades has focused on investigating whether interoceptive deficits are present in individuals with AN and how stable these are.

Based on self‐report, attenuated interoceptive processes in individuals with AN compared with healthy controls (HCs) have been shown consistently (for an overview, see Jenkinson, Taylor, & Laws, 2018). Yet the known limitations of self‐report have driven the search for more objective measures. One psychophysiological indicator of interoceptive processes—accuracy of heartbeat perception (e.g., Schandry, 1981)—yielded more heterogeneous results: Although some studies indeed found lower accuracy in perceiving heartbeats in AN (e.g., Fischer et al., 2016; Pollatos et al., 2008), others did not find evidence for altered heartbeat perception (e.g., Ambrosecchia et al., 2017; Eshkevari, Rieger, Musiat, & Treasure, 2014). Thus, moderating factors might contribute to such heterogeneity.

Conceptually, intuitive eating—referring to eating based on physiological hunger and satiety cues—is strongly tied to interoceptive processes (Tylka, 2006). Similarly, higher heartbeat perception accuracy has been shown to correlate with higher intuitive eating (Herbert, Blechert, Hautzinger, Matthias, & Herbert, 2013). Consistent with this, individuals with AN—who consistently ignore physiological signs of hunger—showed less intuitive eating compared with HCs (van Dyck, Herbert, Happ, Kleveman, & Vögele, 2016). Thus, both interoceptive sensitivity and intuitive eating might contribute to a flexible and balanced eating behavior and both show negative associations with body mass index (BMI) in non‐underweight samples (Herbert et al., 2013; Herbert & Pollatos, 2014).

Weight restoration is the main aim in inpatient treatment for AN. Based on self‐reports, improvements in interoceptive awareness (Preyde, Watson, Remers, & Stuart, 2016) and intuitive eating (Richards, Crowton, Berrett, Smith, & Passmore, 2017) during treatment have been found as well. Therefore, it may be that a higher interoceptive sensitivity and a higher intuitive eating relate to weight gain in AN and, thus, to treatment progress. Such relationships would explain previous inconsistent results: When interoceptive deficits are not stable but improve in the course of inpatient treatment, this may blur group differences in cross‐sectional studies. To date, only one study has examined heartbeat perception accuracy in patients with AN using multiple measurement points and found that reduced interoceptive sensitivity did not improve in the course of inpatient treatment (Fischer et al., 2016). However, this finding was based on only 15 AN patients of restrictive type and, thus, does not provide conclusive evidence that interoceptive deficits in AN are temporally stable.

On the basis of previous findings, we expected that AN patients would show lower interoceptive sensitivity and intuitive eating than HCs. Furthermore, interoceptive sensitivity was expected to correlate positively with higher intuitive eating. Higher interoceptive sensitivity and intuitive eating relate to lower BMI in non‐underweight individuals, suggesting that perceiving bodily signals well and eating in line with these signals help to keep or attain a healthy weight. As individuals with AN are underweight, however, this implies that higher interoceptive sensitivity and intuitive eating would relate to *higher* (i.e., healthier) BMI in patients with AN. Therefore, we expected that higher interoceptive sensitivity and higher intuitive eating would relate to weight gain in AN patients, reflecting improvements in all three variables in the course of treatment.

## 2 METHODS

### 2.1 Participants

Fourty‐four female AN patients and 42 female HCs were interviewed with a structured clinical interview (Saß, Wittchen, & Zaudig, 2003) and the Eating Disorder Examination (Hilbert & Tuschen‐Caffier, 2016) for DSM‐5. Patients met DSM‐5 criteria for AN and were receiving inpatient treatment at the Schoen Clinic Roseneck in Prien am Chiemsee, Germany. Comorbidities included current major depression (*n* = 18), obsessive compulsive disorder (*n* = 9), posttraumatic stress disorder (*n* = 9), social phobia (*n* = 4), bipolar disorder (*n* = 1), generalized anxiety disorder (*n* = 1), and borderline personality disorder (*n* = 1). Sixteen patients received psychopharmacological treatment (selective serotonin reuptake inhibitors [*n* = 7], atypical neuroleptics [*n* = 6], tricyclic antidepressants [*n* = 2], serotonin–norepinephrine reuptake inhibitors [*n* = 1], and norepinephrine–dopamine reuptake inhibitors [*n* = 1]). Some patients were recruited soon after admission (minimum of 3 days), whereas others participated rather late during the course of inpatient treatment (maximum of 130 days) with an average of 49.8 days (*SD* = 37.3) since admission. Mean BMI at admission was 14.2 kg/m^2^ (*SD* = 1.99; range: 10.0–17.6). HCs were recruited and tested with identical routines at the University of Salzburg, Austria. They reported no current or lifetime psychiatric disorders. Exclusion criteria were cardiovascular diseases, psychotic symptoms, substance abuse, and skin allergies. Data from 10 participants were discarded because of technical problems (*n* = 4), skin allergies (*n* = 1), psychological stress (*n* = 2), or because participants did not attend the second laboratory session (*n* = 3), resulting in a sample of 37 patients (78.4% restrictive type, 21.6% binge‐eating/purging type) and 39 HCs.

### 2.2 Measures

#### 2.2.1 Intuitive Eating Scale–2 (IES–2)

The German version of the IES–2 (Tylka & Kroon Van Diest, 2013; van Dyck et al., 2016) was used for assessing intuitive eating. The scale consists of 23 items scored on a 5‐point scale (ranging from 1 [*strongly disagree*] to 5 [*strongly agree*]). Higher scores indicate higher levels of intuitive eating. Internal consistency was α = .867 in the current study.

#### 2.2.2 Heartbeat perception task

A heartbeat perception task (Schandry, 1981) was used for assessing interoceptive sensitivity. Participants first practiced counting their heartbeats silently (without taking their pulse or counting seconds) during a 25‐s time interval. The main task consisted of six time intervals with varying length of 25, 35, 45, 55, 65, and 75 s (order was random across participants). Participants who did not sense any heartbeats were instructed to report zero heartbeats. The appearance/disappearance of a fixation cross marked the beginning/ending of each interval. After each interval, participants were asked to indicate how many heartbeats they had counted. The task was programmed using E‐Prime 2.0 (Psychology Software Tools, Inc., Pittsburgh, PA, United States). Participants were seated at a distance of 50 cm to a 23‐in. LCD monitor.

Interoceptive sensitivity was analyzed by transforming the six time intervals (*n*) according to the following formula:

$$\frac{1}{n}\;\sum_{i=1}^{n}1–\frac{\left|{\text{recorded heartbeats}}_{i}-{\text{counted heartbeats}}_{i}\right|}{{\text{recorded heartbeats}}_{i}}.$$

Scores can range between 0 and 1, with higher scores indicating higher interoceptive sensitivity (i.e., lower discrepancy between recorded and counted heartbeats). Internal consistency across the six time intervals was α = .937.

### 2.3 Electrocardiogram (ECG) recording

For the ECG, two disposable 30 × 24 mm solid‐gel snap electrodes were applied on the upper sternum and distal end of the left costal arch. A 32‐channel amplifier (TMSi, Twente Medical Systems International, EJ Oldenzaal, The Netherlands) and Polybench 1.3 (TMSi) were used for recording the ECG. Signals were digitized with a 512‐Hz sampling rate. Data were inspected offline with ANSLAB 2.6 (Blechert, Peyk, Liedlgruber, & Wilhelm, 2016). Artifacts were corrected manually, and the program determined the number of heartbeats in each time interval by counting the number of R‐spikes.

### 2.4 Procedure

The study was approved by the medical review board of the University of Munich, Germany, and all participants (and—when underaged—also their parents) provided written informed consent. At a first laboratory session, participants were interviewed via structured clinical interviews by the same trained interviewer for exclusion criteria and current/past psychiatric disorders (including affective disorders, anxiety disorders, psychotic disorders, obsessive–compulsive disorders, dissociative disorders, eating disorders, and borderline personality disorder). Afterwards, participants completed the IES–2 among other questionnaires. At a second laboratory session, ECG electrodes were attached, and participants completed the heartbeat perception task, followed by other experimental paradigms not reported here.

### 2.5 Data analyses

Group differences in age, years of education, BMI, interoceptive sensitivity, intuitive eating, and heart rate were tested with independent sample's *t* tests. Next, a correlation between interoceptive sensitivity and intuitive eating was calculated. However, as this correlation may be different in the two groups, we tested for group differences in correlation strength with a linear regression analysis with interoceptive sensitivity, group, and their interaction term as independent variables and intuitive eating as dependent variable. Finally, correlations of interoceptive sensitivity and intuitive eating with BMI and days since admission were calculated. As participants with AN were currently gaining weight during their inpatient treatment, current BMI was controlled for BMI at admission through partial correlations to index treatment progress.

## 3 RESULTS

### 3.1 Group differences

Groups did not differ in age, years of education, heart rate, and interoceptive sensitivity. Patients with AN had lower BMI and lower intuitive eating than HCs (Table 1).

**Table 1:** Descriptive statistics with means (M), standard deviations (SD), and range for patients with anorexia nervosa and healthy controls

	Patients with anorexia nervosa (n = 37)			Healthy controls (n = 39)			Test statistics
	M	SD	Range	M	SD	Range	
Age (years)	22.5	5.06	17.0–35.0	22.0	3.55	16.0–35.0	*t*(64.1) = 0.54, *p* = .590, *d* = 0.12
Education (years)	14.8	3.06	10.0–22.0	14.6	2.30	11.0–21.0	*t*(74) = 0.27, *p* = .786, *d* = 0.07
Body mass index (kg/m^2^)	15.4	1.80	12.8–18.2	21.8	1.76	19.0–24.5	*t*(74) = −15.5, *p* < .001, *d* = −3.60
Intuitive Eating Scale–2	2.78	0.59	1.60–4.00	3.64	0.43	2.50–4.30	*t*(74) = −7.30, *p* < .001, *d* = −1.67
Heart rate (beats/minute)	74.5	8.76	54.0–89.2	73.7	9.68	52.2–95.0	*t*(74) = 0.39, *p* = .700, *d* = 0.09
Interoceptive sensitivity	0.46	0.22	0.00–0.83	0.53	0.22	0.00–0.91	*t*(74) = −1.40, *p* = .165, *d* = −0.32

### 3.2 Relationship between interoceptive sensitivity and intuitive eating

Higher interoceptive sensitivity related to higher intuitive eating (*r* = .269, *p* = .019). This association was independent of group as indicated by a nonsignificant interaction term in the regression analysis (*b* = 0.66, *SE* = 0.53, *p* = .217).

### 3.3 Relationships of interoceptive sensitivity and intuitive eating with BMI and treatment progress

**Figure 1:**
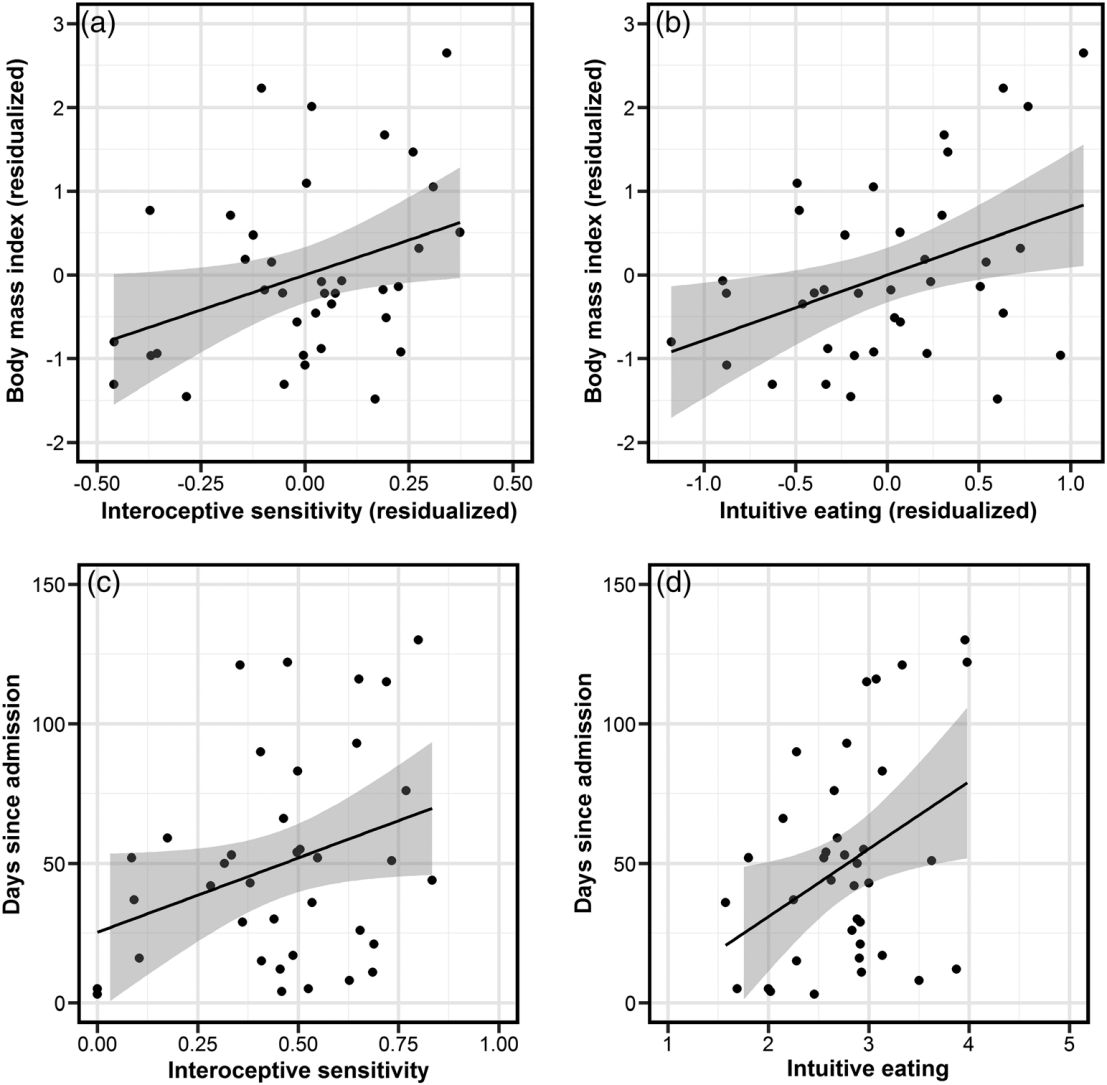
Scatterplots depicting correlations of body mass index with (a) interoceptive sensitivity and (b) intuitive eating (when partialling out body mass index at admission) and of days since admission with (c) interoceptive sensitivity and (d) intuitive eating in patients with anorexia nervosa. As panels (a) and (b) represent partial correlations, residualized scores of interoceptive sensitivity, intuitive eating, and body mass index are displayed. Grey areas denote 95% confidence intervals

In HCs, BMI did not correlate with interoceptive sensitivity (*r* = −.138, *p* = .400) or intuitive eating (*r* = −.139, *p* = .400). In patients with AN, a higher BMI (when controlling for BMI at admission) related to higher interoceptive sensitivity (*r* = .351, *p* = .018; Figure 1a) and higher intuitive eating (*r* = .405, *p* = .007; Figure 1b). Furthermore, days since admission related to higher BMI (when controlling for BMI at admission, *r* = .832, *p* < .001), higher interoceptive sensitivity at a trend level (*r* = .320, *p* = .054; Figure 1c), and higher intuitive eating (*r* = .380, *p* = .020; Figure 1d).

## 4 DISCUSSION

The aim of the current study was to investigate whether interoceptive deficits are present in individuals with AN with respect to the accuracy of perceiving cardiac activity (i.e., counting heartbeats). We further examined relationships with intuitive eating, BMI, and number of days since admission for gaining a better understanding of the effects of treatment progress on these variables. In line with previous studies (van Dyck et al., 2016), AN patients reported lower intuitive eating than HCs in the current study. Moreover, higher intuitive eating related to higher interoceptive sensitivity, confirming their conceptual relationship (Herbert et al., 2013) and extending it to a sample ranging from extreme underweight and very low intuitive eating to normal weight and normal intuitive eating levels. This suggests that perceiving bodily signals relates positively to reports of eating according to these signals and supports the validity of the concept of intuitive eating. Furthermore, it suggests that interoception is a general process that cuts across modalities (e.g., cardiac to gastric modalities; Herbert, Muth, Pollatos, & Herbert, 2012).

In contrast to previous reports (Fischer et al., 2016; Pollatos et al., 2008), but in line with others (Ambrosecchia et al., 2017; Eshkevari et al., 2014), we did not find evidence for lower heartbeat perception in AN patients compared with HCs using a heartbeat perception task. This may be due to sample differences (e.g., inpatient vs. outpatient, smaller sample sizes, or presence of comorbidities) and resulting heterogeneity in weight and treatment progress. Whereas we tested 37 AN patients (~80% of restrictive type) at different stages of treatment, Fischer et al. (2016) tested 15 AN patients with a restrictive type at three relatively systematic measurement points. Furthermore, Pollatos et al. (2008) recruited 28 AN outpatients (~80% of restrictive type), suggesting that treatment progress may be of importance when investigating interoceptive deficits in AN. It may be, for example, that interoceptive deficits are only present prior to or at the beginning of inpatient treatment and/or in individuals with extremely low BMI.

This interpretation is supported by considering associations with weight gain and days in the hospital in the current study: Higher interoceptive sensitivity was found in patients who had gained more weight and had been in the hospital for a longer time. Thus—although based on cross‐sectional data—the current results indicate that interoceptive processes may be influenced by state‐dependent factors and heterogeneity in treatment progress. This contrasts with findings of Fischer et al. (2016), who did not find evidence for improvements of interoceptive deficits in the time course of treatment. Of note, however, is that the study by Fischer et al. (2016) had relatively low power, and descriptively, it appeared that interoceptive sensitivity did indeed improve in AN patients and this increase might have been significant in a larger sample. Future longitudinal studies may, therefore, include multiple and systematic follow‐up measurement points and replicate findings of Fischer et al. (2016) in larger samples in the time course of inpatient treatment for investigating interoceptive deficits in AN.

As the current study relied on cross‐sectional data, inferring causal directions is not possible: It may be either that higher interoceptive sensitivity and higher intuitive eating promote weight gain or that improvements in interoceptive sensitivity and intuitive eating are a result of weight gain. Furthermore, Wittkamp, Bertsch, Vögele, and Schulz (2018) argued that at least two measurement points should be applied in order to interpret interoceptive processes as a trait. Thus, when interoceptive processes are measured only once—which is common practice—state‐dependent variables (e.g., BMI, treatment duration) may affect whether or not interoceptive deficits are observable in AN.

Finally, previous research suggests that depression and anxiety are negatively related to heartbeat perception in healthy samples (e.g., Pollatos, Traut‐Mattausch, & Schandry, 2009). As the current sample of AN patients had a high rate of comorbid mental disorders, it may be that (changes in) interoceptive awareness may be influenced by the presence of these disorders as well. Thus, the question of whether AN patients with comorbid affective or anxiety disorders differ from AN patients without these disorders is an open and certainly fruitful future direction. Furthermore, the role of pharmacological treatment on heartbeat perception accuracy has received relatively little attention, as participants undergoing pharmacological treatment are usually either omitted (e.g., in Fischer et al., 2016) or—as in the current study—sample sizes are too small to systematically examine effects of pharmacological medication.

In conclusion, the current findings indicate that interoceptive deficits may be influenced by treatment progress (i.e., BMI change and time since admission). What follows from this is that cross‐sectional studies will probably obtain different findings, depending on treatment stage of included AN patients. The wider literature actually suggests that the relationships of interoceptive sensitivity and intuitive eating with body weight may be non‐linear and follow an inverted U‐shaped function: Both interoceptive sensitivity and intuitive eating seem to be negatively related to body weight in non‐underweight individuals (Herbert et al., 2013; Herbert & Pollatos, 2014), but are positively related to body weight (recovery) in individuals with AN. Thus, it appears that both increased interoceptive sensitivity and intuitive eating can contribute to a healthy weight in all individuals (i.e., increasing body weight in underweight individuals and decreasing body weight in overweight individuals towards a normal weight).
